# Demonstration of measuring sea fog with an SNSPD-based Lidar system

**DOI:** 10.1038/s41598-017-15429-y

**Published:** 2017-11-08

**Authors:** Jiang Zhu, Yajun Chen, Labao Zhang, Xiaoqing Jia, Zhijun Feng, Ganhua Wu, Xiachao Yan, Jiquan Zhai, Yang Wu, Qi Chen, Xiaoying Zhou, Zhizhong Wang, Chi Zhang, Lin Kang, Jian Chen, Peiheng Wu

**Affiliations:** 1 Research Institute of Superconductor Electronics, Nanjing University, Nanjing, 210093 China; 2 Nanjing Research Institute of Electronic Technology, Nanjing, 210039 China; 3 Zhongyuan Research Institute of Electronic Technology, Zhengzhou, 450047 China

## Abstract

The monitor of sea fogs become more important with the rapid development of marine activities. Remote sensing through laser is an effective tool for monitoring sea fogs, but still challengeable for large distance. We demonstrated a Long-distance Lidar for sea fog with superconducting nanowire single-photon detector (SNSPD), which extended the ranging area to a 180-km diameter area. The system, which was verified by using a benchmark distance measurement of a known island, is applied to the Mie scattering weather prediction Lidar system. The fog echo signal distribution in the range of 42.3∼63.5 km and 53.2∼74.2 km was obtained by the Lidar system. Then the fog concentration and the velocity of the fog were deduced from the distribution, which is consistent with the weather prediction. The height of the sea fog is about two hundred meter while the visibility at this height is about 90 km due to the Earth’s radius of curvature. Therefore, the capability of this SNSPD-based Lidar was close to the theoretical limit for sea fog measurements for extremely high signal-to-noise ratio of SNSPD.

## Introduction

Sea fog is a common phenomenon of sea weather and has a significant impact on fisheries, navigation, and flight safety. Laser weather radar^1^ is a commonly used sea fog detection technology with excellent azimuth accuracy, distance resolution, and high—quality, low background noise. Laser photon radar based on single photon detectors is used to detect the range and concentration of clouds, humidity, wind fields, air pollution, pollutant diffusion, and to provide weather forecasts^2,3^.

At present, the single photon detectors used in lidar weather prediction are mainly InGaAs/InP avalanche photodiodes operated in the Geiger mode^4,5^. In order to improve the detection rate, active, passive, gate pulse suppression, and other methods are used for self-sustaining avalanche quenching. The infrared detection efficiency is often less than 30% and the dark counts are a few kHz^6^. Thus, traditional Lidar for weather prediction was around twenty kilometer range. SNSPD is a new type of single photon detector^7–13^ with high detection efficiency, low dark count, fast detection rate, high sensitivity, wide response spectrum, and other advantageous features^14,15^. The detection efficiency is up to 93% in the near infrared band while the dark count is less than 100 Hz. SNSPD has been applied to distance measurements based on a satellite laser system^16,17^. But to date, the technology has not been applied to sea fog measurements. In the detection of remote atmospheric particles, the echo signal is extremely weak and the detector signal to noise ratio requirement is extremely high. Therefore, the high efficiency, low noise SNSPD system has obvious advantages for long-distance sea fog detection.

In this study, SNSPD is used to explore the influence of the distance to the fog on the echo signal rate. The distribution and range of the fog are calculated and the results show that the SNSPD proved capable to detect this long-distance meteorological phenomenon.

## Materials and Methods

### Atmospheric detection principle

According to Lidar theory, the particle scattering follows the Mie scattering theory when the incident wavelength $\lambda$ is much smaller than the radius r of the detected particle (*i.e*., $(i\mathrm{.}e\mathrm{.,}\lambda \ll r)$. Mie scattering laser radar has been used to detect aerosol particles such as dust and clouds in the low altitude atmosphere below 30 km and has proven to be useful for understanding the characteristics and effects of aerosol particles and the subsequent pollution control.

In experiments, cloud droplets, raindrops, droplets, and ice crystals shape are usually assumed to have a spherical shape. Sea fog is mostly advection fog, the radius of the droplets is generally in the range of 3~60 μm, and the mode average radius is about 7 μm. The laser emission wavelength is 1064 nm and for the spherical particles, *α* = 2 $\pi$ r/$\lambda$ = 41.335,which satisfies $\lambda \ll {\rm{r}}$; therefore, the scattering of light by the droplets follows the Mie scattering theory. The scattering of the particles in all directions can be expressed by the scattering function *β*($\lambda$, R, ${\rm{\Theta }}$)(R represents the distance of targets). When ${\rm{\Theta }}$ = $\pi$, the backscattering function of the particle $\beta$($\lambda$, R, $\pi$)is expressed as follows:

1 $$\beta (\lambda ,R,\pi )=\frac{{\lambda }^{2}}{16{\pi }^{2}}{|{\sum }_{n=1}^{\infty }{(-1)}^{n}(2n+1)({a}_{n}-{b}_{n})|}^{2}$$

where a_*n*_, b_*n*_ are the scattering field coefficients. The backscattering cross section $\sigma$($\lambda$) = 4 $\pi$ $\beta$($\lambda$, R, $\pi$) is described as follows:

2 $${\rm{\sigma }}(\lambda )=\frac{{\lambda }^{2}}{4\pi }{|{\sum }_{n=1}^{\infty }{(-1)}^{n}(2n+1)({a}_{n}-{b}_{n})|}^{2}$$

### Fog concentration formula

The signal strength of the Lidar echo is affected by three types of parameters: (1) the reflection intensity of the measured object; (2) the attenuation of the atmosphere; (3) the configuration and performance of the laser radar’s transmission and receiving system. Therefore, the equation for the atmospheric lidar detection is^18^:

3 $$P(R)=\mu \cdot {P}_{0.}\,\frac{A}{{R}^{2}}\cdot {\rm{\Delta }}R\cdot \beta (\lambda ,R)\exp [-2{\int }_{0}^{R}\alpha (\lambda ,R)dR]$$

where P(R) is the echo signal power from Lidar receiving distance R to R + ${\rm{\Delta }}$ R, *μ* is the correction constant of the Lidar, P_0_ is the instantaneous transmission power, R is the detection distance, A is the area of the receiving telescope, ${\rm{A}}=\frac{\pi {D}^{2}}{4}$, $\beta$($\lambda$, R)is the backscattering coefficient of the particles in the atmosphere, and *α*($\lambda$, R) is the total extinction coefficient of the atmosphere. ${\rm{C}}\cdot {{\rm{P}}}_{0}\frac{A}{{R}^{2}}\cdot {\rm{\Delta }}\,{\rm{R}}$ is the radar parameter and is the only parameter relevant to the Lidar and $\beta (\lambda ,R)\exp [-2{\int }_{0}^{R}\alpha (\lambda ,{\rm{R}})\mathrm{dR}]$ is the only parameter relevant to the atmosphere. The formula derived for this experiment is:

4 $$n=\frac{\lambda }{{h}_{c}}\cdot {P}_{t}\cdot \frac{{\rm{\Delta }}\tau \cdot c}{8}\cdot \frac{{\mu }_{t}\cdot {\mu }_{r}\cdot {\mu }_{d}\cdot {\mu }_{s}\cdot \pi \cdot {D}^{2}}{{R}^{2}}\cdot \beta (\lambda ,R)\cdot \exp [-2{\int }_{0}^{R}\alpha (\lambda ,R)dR]$$

where n is the number of single-pulse echo photons; $\lambda$ is the wavelength of the emission laser, 1064 nm; h is the Planck constant, 6.626 × 10^−34^ J s; c is the speed of light, 3 × 10^8^ m/s; P_*t*_ is the emission laser peak power; ${\rm{\Delta }}$ $\tau$ is the emission laser pulse width; D is the diameter of the receiving telescope; ${\mu }_{t}$ is the emission optical efficiency; ${\mu }_{r}$ is the receiving optical system efficiency; ${\mu }_{d}$ is the detector efficiency, 80%; and ${\mu }_{s}$ is the system correction coefficient.

$\beta$($\lambda$, R) is the backscattering coefficient that represents the scattering energy of the laser beam in the atmosphere at a distance R, $\beta (\lambda ,R)=N(R)\cdot {\rm{\sigma }}(\lambda )$, in which $\sigma$($\lambda$) is the backscattering cross section for a detected particle, cm^2^ sr^−1^; and N(R) is the detected particle density, cm^−3^. $\beta$($\lambda$, R)indicates that the intensity of the echo signal is proportional to the backscattering cross section and density of the particle being detected. Hence, the detected particle density can be derived from the strength of the echo signal as follows:

5 $$N(R)=n\cdot \frac{h}{\lambda }\cdot \frac{{R}^{2}}{{p}_{t}\cdot {\rm{\Delta }}\tau \cdot {\rm{\sigma }}(\lambda )}\cdot \frac{8}{{\mu }_{t}\cdot {\mu }_{r}\cdot {\mu }_{d}\cdot {\mu }_{s}\cdot {D}^{2}\cdot \pi }\cdot {\exp }[{\rm{2}}{\int }_{0}^{R}\alpha (\lambda ,R)dR]$$

### SNSPD system

The automatic control system of the SNSPD consists of an optical coupling system, a cooling system, an output circuit, and a display control system. The preparations for operating the SNSPD consisted of NbN film growth, nanowire preparation, electrode growth, optical resonator growth, packaging, testing, etc. We used MgF_2_ as the substrate to grow the NbN film with a size of 5 nm and the nanowires were created using electron beam lithography (EBL). We also fabricated Au electrode and optical resonant cavity on SNSPD. The SNSPD critical temperature is 8.5 K, the critical current is 7.5 $\mu$A, and the photosensitive area is 10 $\mu$m * 10 $\mu$m. The NbN energy gap is 5.12 meV, which is much smaller than the photon energy (1 eV); therefore, the 100-nm wide nanowires have high detection efficiency in the infrared band. Compared with a Si substrate, the MgF_2_ is more compatible with the NbN lattice constants and has a greater thermal conductivity. Hence, the SNSPD with a substrate of MgF_2_ has a better performance and a higher detection rate. The SNSPD system based on a 62.5-$\mu$m multimode fiber coupling has a quantum detection efficiency of 80% for a wavelength of 1064 nm and a dark count rate (DCR) of less than 100 cps^19,20^. The automatic control system displays the real-time cold head air pressure and temperature during the vacuum and cooling process. When the SNSPD reaches the required temperature, the computer controls the current and voltage, monitors the SNSPD in real time during operations, and detects and sounds alarms for abnormal states.

### Measurement system

**Figure 1:**
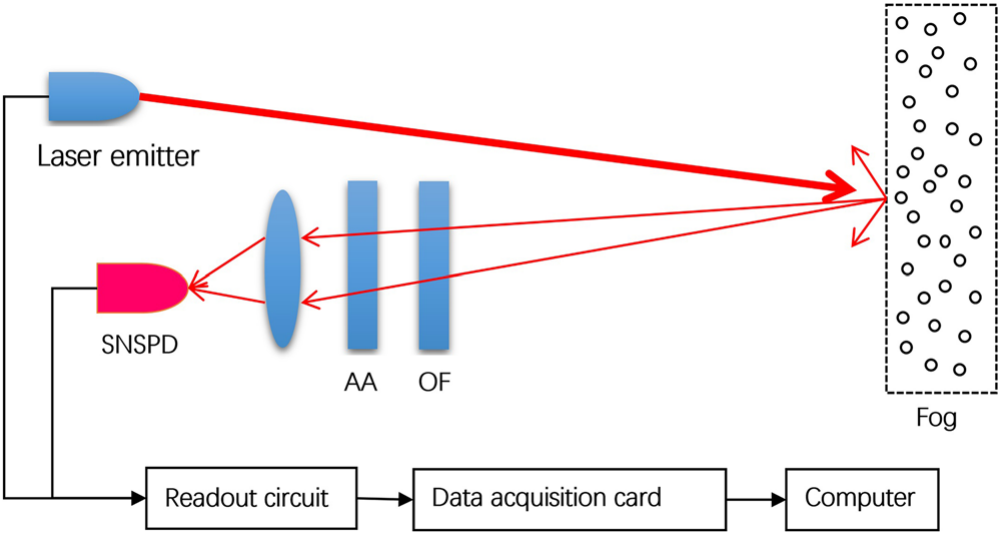
Schematic diagram of the laser measurement system (OF: Optical filter, AA: Adjustable attenuator). When the laser signals are propagated in the atmosphere, cloud, rain, ice, and other suspended particles cause a scattering of the signals. An optical telescope receives the backscattered light and the SNSPD is used to convert the optical signals into electric pulse signals.

The measurement system of Lidar includes a 1064-nm laser transmitter, a receiving telescope, a filter, an optical attenuator, a SNSPD, an output circuit, a data acquisition card, and a computer as Fig. 1 ^21^. The laser emits a pulse with a wavelength of 1064 nm, a frequency of 1 Hz, a pulse width of 10 ns, an emission optical efficiency of 0.85, a laser emission angle of 0.2 mrad, and a laser peak power of 12 MW. The telescope receives the echo signal that is transmitted to the 62.5-$\mu$m multimode fiber with a receiving aperture of 120 mm and a receiving optical efficiency of 0.8. The optical fiber is connected to the adjustable fiber attenuator; the output light passes through the 1064-nm filter that is connected to the photosensitive surface of the SNSPD with an optical microlens. The coupling efficiency is about 90%. When the SNSPD detects a single photon, it generates a voltage pulse signal that is amplified by the output circuit to a voltage of 100 mV. The counter records the number of voltage pulse signals and transmits this information to the computer via a data acquisition card. The filter attenuation, the device insertion loss, and the fiber loss are approximately 4 dB.

## Results and analysis

### Test at Dagong Island

**Figure 2:**
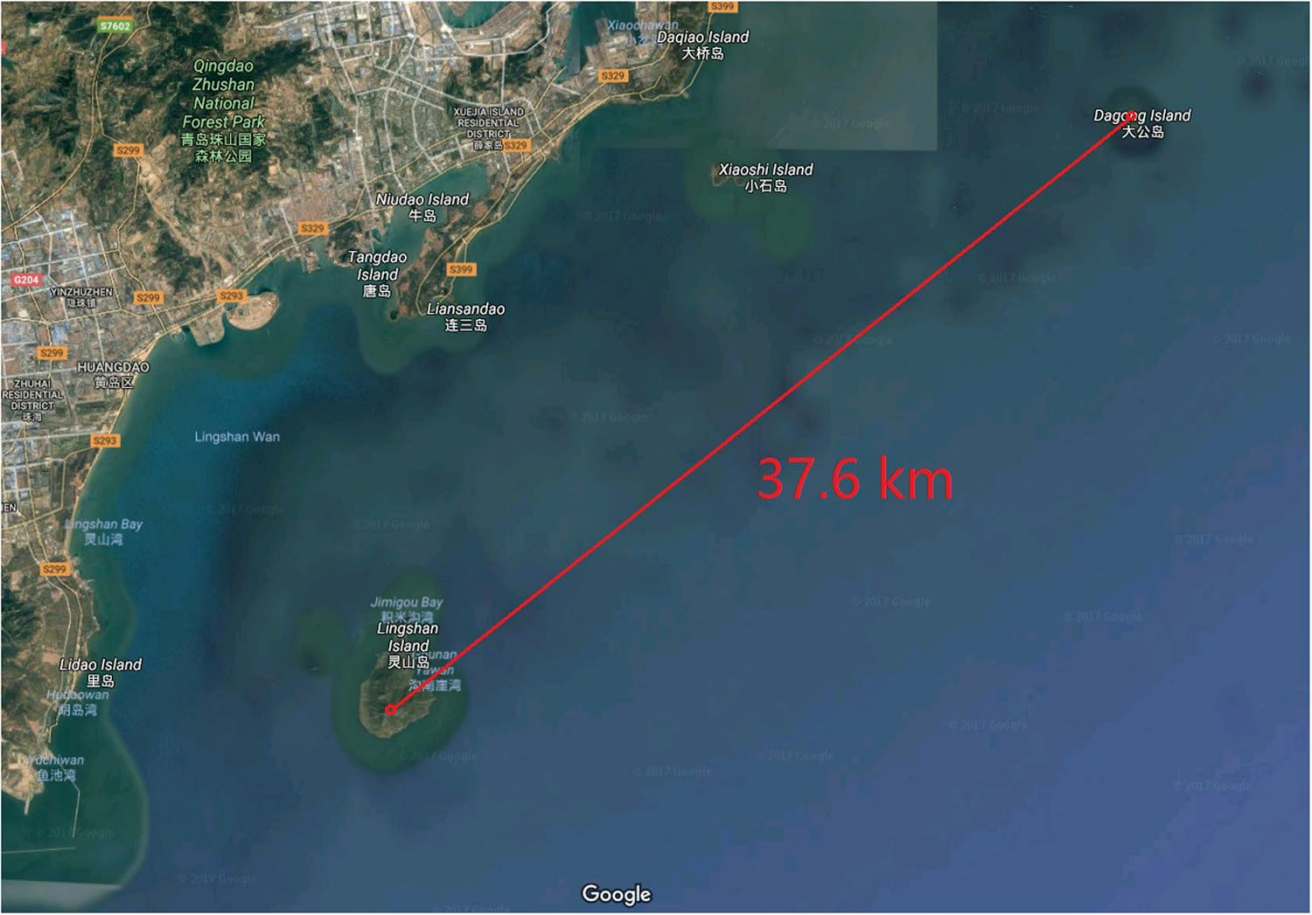
The locations of Dagong Island and Lingshan Island on the Google map. The experimental site is located at a distance of 37.6 km from Dagong Island. (Imagery ©2017 CNES/Airbus, TerraMetrics, Data SIO, NOAA, U.S. Navy, NGA, GEBCO, DigitalGlobe, DigitalGlobe, Map data ©2017 United States. The map was generated using the software Google Earth 7.1.5.1557 (Google Inc. 2017) (https://goo.gl/maps/EitKEXPv6E82).

**Figure 3:**
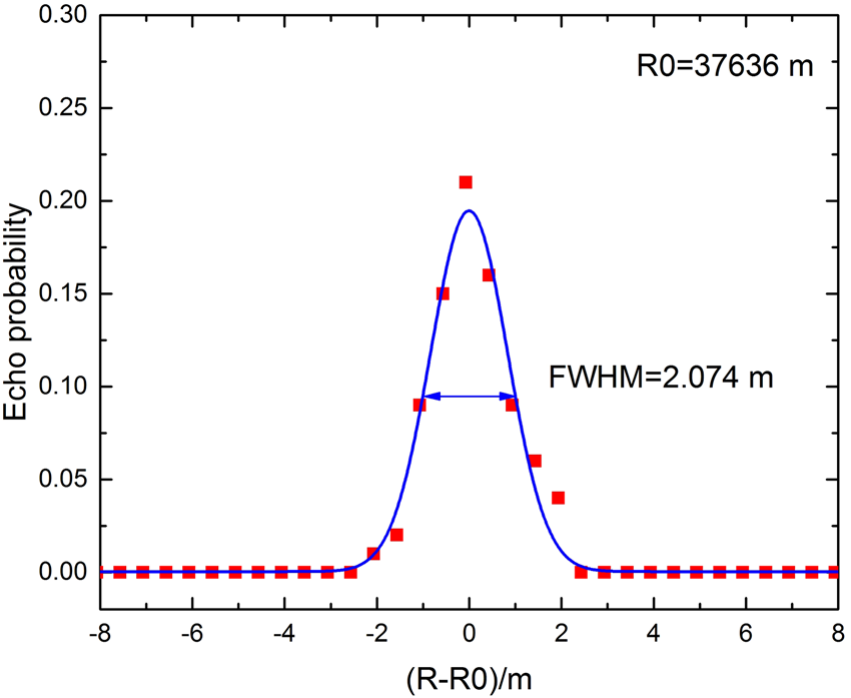
Gaussian fitting for the echo probability of the signal for Dagong Island.

In order to validate the measurement system, we first measured the distance of a building on Dagong Island, which is located at a distance of 37 km. The known distance measurement serves as a benchmark of the system to test the accuracy. The visibility was 23 km and the targeted building on Dagong Island was located in the northeast part of the test site at a distance of 37 km, as shown in Fig. 2. The optimum operating conditions were adjusted by changing the operating current and the attenuator values, and by observing the change in the echo signal. The SNSPD bias current was set to 6.4 ua and the receiver’s attenuator was set to 20 dB. The echo signal exhibited a Gaussian distribution near the target and the fit is shown in Fig. 3. The full width at half maximum (FWHM) of the Gaussian curve represents the system’s distance resolution, FWHM = 2.07 m. The measured distance was 37.636 km, which matches the actual distance (37.6 km) between Lingshan Island and Dagong Island and demonstrates the accuracy of the SNSPD measurement system.

### Sea fog echo signal

**Figure 4:**
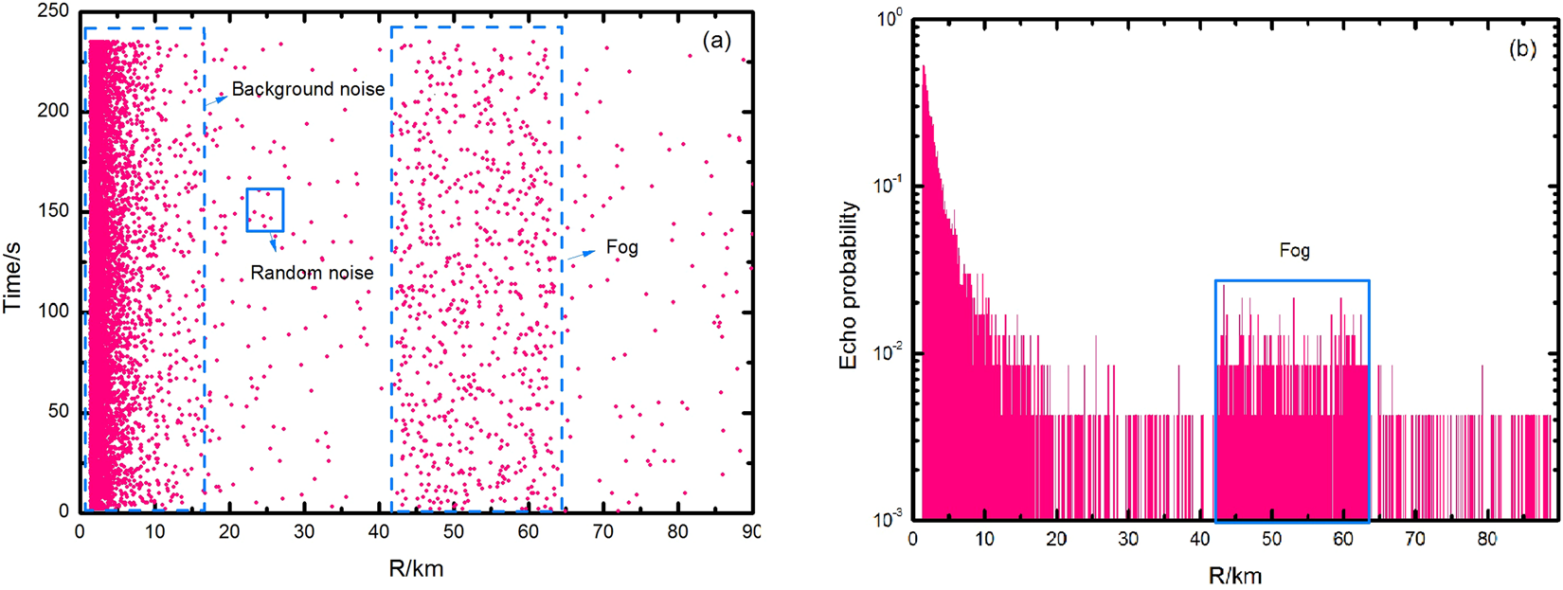
Measurement results for the fog in the range of 42.3~63.5 km: (**a**) experimental data of the echoes; (**b**) results of echo probabilities. The echo signal of the fog, 0.71%, is significantly stronger than the background.

The laser signal is emitted at a frequency of 1 Hz with the altitude of 117 m at different times during the day with a visibility of 23 km. The attenuation is set to 10 dB at the receiver due to strong backscatter and the echo number and echo rate are shown in Fig. 4. The horizontal and vertical axes in (a) are the distance (R/m) and the pulse number respectively while the vertical axis in (b) is the echo rate, which is the average photon number of the SNSPD’s single-pulse response. The sea surface has a strong backscattering due to a high humidity compared to the land surface, and its signal is nearly hidden within 15 km. As shown in Fig. 4(a), the backscattering decays exponentially. The dark count, the background noise, and the circuit noise are relatively small^22^, while the backscatter can be ignored at a distance beyond 20 km. In the range of 42.3~63.5 km, the echo signal is strong where the mist has formed. The maximum echo rate is 2.1%, and the average echo rate is 0.287% (Fig. 4(b)).

**Figure 5:**
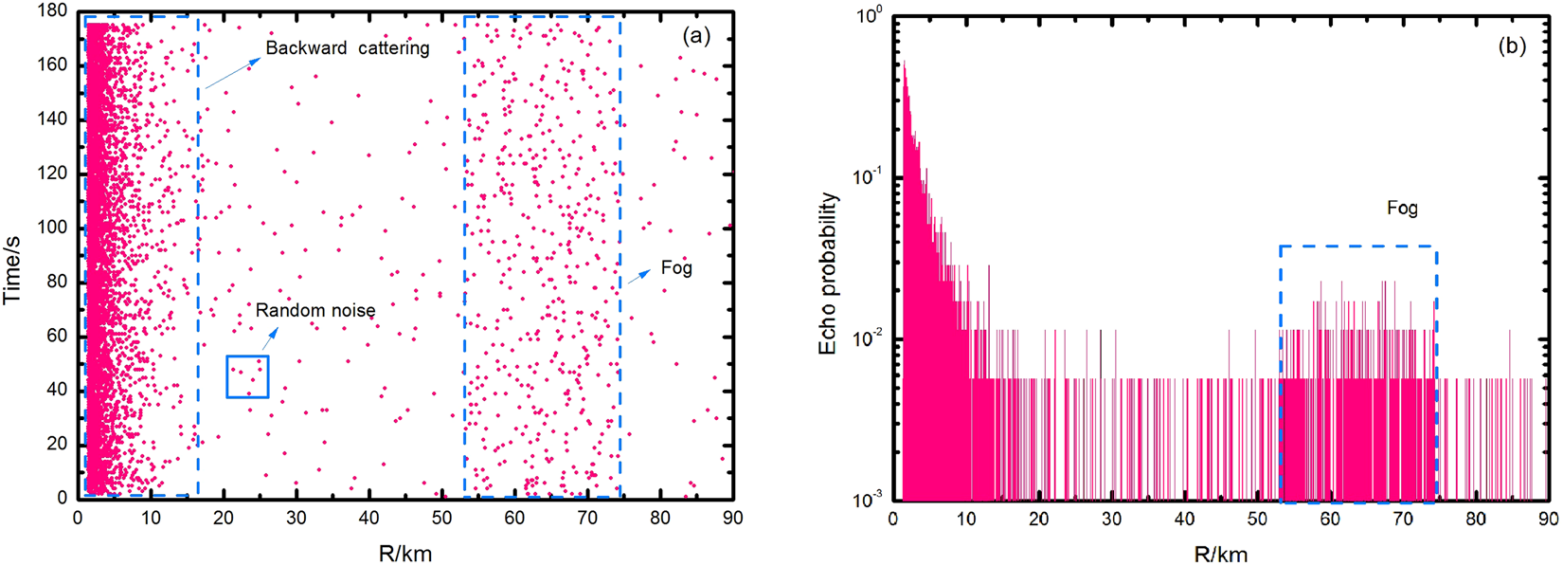
Measurement results for a target in the range of 53.2~74.2 km: (**a**) experimental data of the echoes; (**b**) results of echo probabilities. The echo signal of the fog, 0.57%, is significantly stronger than the background.

The laser was emitted in the same direction and with the same frequency after 45 minutes and the number of received echoes and the echo rate are shown in Fig. 5(a) and (b). The echo rate has increased in the distance range of 53.2~74.2 km. As shown in Fig. 5(b), the peak echo rate is 2.3%, the mode is 0.57%, and the average echo rate is 0.267% at a range of 21.0 km. The fog was an advection fog. The velocity of the fog along the laser’s emission direction was calculated as 13 km/h, which matched the real-life situation^22^.

### Fog movement

The empirical relationship between the atmospheric extinction coefficient *α*, the laser wavelength $\lambda$, and the atmospheric visibility S is as follows:

6 $$\alpha (\lambda ,R)=\frac{3.91}{S}(\frac{0.55}{\lambda })q$$

where the atmospheric extinction coefficient *α* has a unit of km, the laser wavelength $\lambda$ has a unit of *μ*m, and the atmospheric visibility S has a unit of km. In the experiment, the atmospheric visibility was 35 km, q was 1.3, the atmospheric extinction coefficient $\alpha$ was 0.047, the humidity was 72%, the temperature was 10 °C, the fog droplet reflectivity m was 1.33, and ${\rm{\sigma }}$($\lambda$) was 5 × 10^−16^ cm^2^/sr. Hence, the fog concentration was:

7 $$N(n,R)=\frac{1.687\times {10}^{-22}}{{\rm{\sigma }}(\lambda )}\times n{R}^{2}{e}^{0.09R}$$

8 $$N(n,R)=3.37\times {10}^{-7}\times n{R}^{2}{e}^{0.09R}$$

The fog was assumed to be uniform due to the existence of the extinction coefficient of the fog ${\alpha }_{m}$. In order to ignore the influence of the extinction coefficient, the distance R_1_ was 45 km, n_1_ = 0.71% (from Fig. 4), and the distance from R_2_ was 55 km, n_2_ = 0.57% (from Fig. 5). Thus, the fog concentration was N_1_ = 278 cm^−3^ in Fig. 4 and N_2_ = 820 cm^−3^ in Fig. 5 calculated by Eq. 8). The fog was moving and the concentration increased significantly over time.

**Figure 6:**
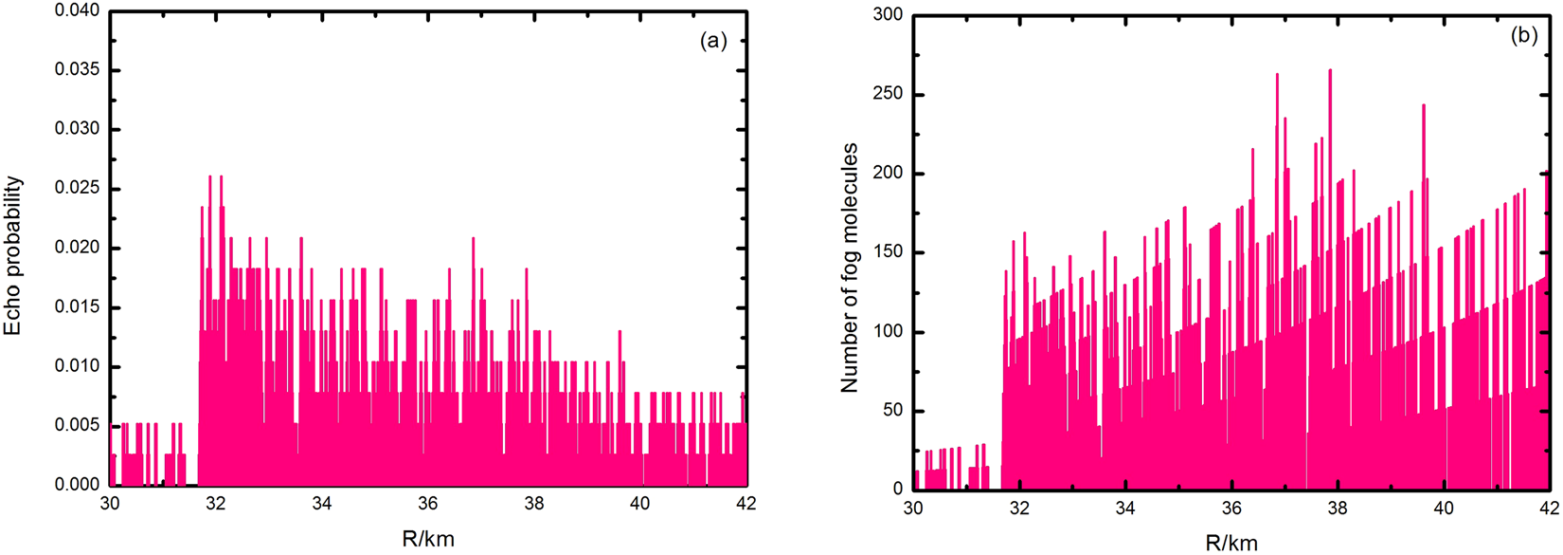
Measurement results for the target in the range of 31.6~42.0 km: (**a**) results of echo probability. The graph shows that the echo signal decreases with an increase in the distance; (**b**) number of fog molecules. The fog concentration is dense in the center and less dense at the edges.

The echo signal was measured in the opposite direction (from a distance of 31.6 km to 42.0 km) to measure the distribution of the fog in more detail and verify the feasibility and accuracy of the SNSPD-based Lidar. Meteorological measurements was applied to predict with the limitation of the Lidar system. Figure 6(a) shows the echo signal rate based on the fog distribution values derived from Fig. 6(b). In the range of 36.6~37.8 km, the maximum fog concentration is 265 cm^−3^ and the fog concentration is dense in the center and less dense at the edges. At present, a droplet spectrometer is used to measure the fog concentration, which can be also used to measure the distribution of the concentration, the number of concentration and the effective diameter of droplets. However, this approach requires too much time to be used for long-distance fog measurements. Meteorological stations do not predict sea fog due to technical restrictions. The measured data in this study were compared with the results 248.9 cm^−3^ from studies by Xu, *et al*.^23^ and others that used a “Multiple-use drop spectrometer” to measure the fog concentration in Maidao, which is located close to Qingdao. This result is consistent with Xu’s conclusions.

## Conclusions

In this study, the echo signal distribution in a 180-km diameter area is measured and the fog echo signals in the ranges of 42.3~63.5 km and 53.2~74.2 km are detected using an SNSPD lidar system. The fog concentrations at distances of 45 km and 55 km are 278 cm^−3^ and 820 cm^−3^ respectively at different times and the fog’s velocity along the direction of the laser emission is 13 km/h. Furthermore, the echo signal in the range of 31.6~42.0 km is also detected and the fog distribution is derived based on the formula for the fog concentration. The fog concentration at a range of 31.6~42.0 km is 150 cm^−3^ and the maximum value of the fog concentration is 265 cm^−3^. The fog concentration is dense in the center and less dense at the edges, which is consistent with the characteristics of the fog distribution. These results prove that the SNDD-based lidar can effectively improve the measurements of the detection distance and the detection accuracy compared with the ~20 km of traditional Lidar system. In addition, the SNSPD array devices that are being developed have high detection efficiency, quantitative resolution, and position resolution. A wide application of the SNSPD array devices will improve the accuracy of long-distance measurements applied to meteorological phenomena.
